# *ABCA7* polymorphisms correlate with memory impairment and default mode network in patients with *APOE*ε4-associated Alzheimer’s disease

**DOI:** 10.1186/s13195-019-0563-3

**Published:** 2019-12-12

**Authors:** Ya-Ting Chang, Shih-Wei Hsu, Shu-Hua Huang, Chi-Wei Huang, Wen-Neng Chang, Chia-Yi Lien, Jun-Jun Lee, Chen-Chang Lee, Chiung-Chih Chang

**Affiliations:** 1 Department of Neurology, Institute of translational research in biomedicine, Kaohsiung Chang Gung Memorial Hospital, Chang Gung University College of Medicine, 123, Ta-Pei Road, Niaosung, Kaohsiung, 833 Taiwan; 2 Department of Radiology, Kaohsiung Chang Gung Memorial Hospital, Chang Gung University College of Medicine, 123, Ta-Pei Road, Niaosung, Kaohsiung, 833 Taiwan; 3 Department of Nuclear Medicine, Kaohsiung Chang Gung Memorial Hospital, Chang Gung University College of Medicine, 123, Ta-Pei Road, Niaosung, Kaohsiung, 833 Taiwan

**Keywords:** Alzheimer’s disease, *APOE*, Default mode network, Genetics, Memory

## Abstract

**Background:**

Since both APOE and *ABCA7* protein expression may independently reduce neuritic plaque burden and reorganize fibrillar amyloid burden-mediated disruption of functional connectivity in the default mode network, we aimed to investigate the effect of the *APOE-ABCA7* interaction on default mode network in Alzheimer’s disease.

**Methods:**

Two hundred and eighty-seven individuals with a diagnosis of typical Alzheimer’s disease were included in this study. Memory was characterized and compared between *APOE*-ε4+ carriers and *APOE*-ε4 non-carriers within *ABCA7* rs3764650T allele homozygous carriers and *ABCA7* rs3764650G allele carriers, respectively. Two-way analysis of variance was used to identify a significant interaction effect between *APOE* (*APOE*-ε4+ carriers versus *APOE*-ε4 non-carriers) and *ABCA7* (*ABCA7* rs3764650T allele homozygous versus *ABCA7* rs3764650G allele carriers) on memory scores and functional connectivity in each default mode network subsystem.

**Results:**

In *ABCA7* rs3764650G allele carriers, *APOE*-ε4+ carriers had lower memory scores (*t* (159) = − 4.879; *P* < 0.001) compared to *APOE*-ε4 non-carriers, but *APOE*-ε4+ carriers and *APOE*-ε4 non-carriers did not have differences in memory (*P* > 0.05) within *ABCA7* rs3764650T allele homozygous carriers. There was a significant *APOE-ABCA7* interaction effect on the memory (F3, 283 = 4.755, *P* = 0.030). In the default mode network anchored by the entorhinal seed, the peak neural activity of the cluster that was significantly associated with *APOE*-*ABCA7* interaction effects (*P* = 0.00002) was correlated with the memory (*ρ* = 0.129, *P* = 0.030).

**Conclusions:**

Genetic-biological systems may impact disease presentation and therapy. Clarifying the effect of *APOE-ABCA7* interactions on the default mode network and memory is critical to exploring the complex pathogenesis of Alzheimer’s disease and refining a potential therapy.

## Background

*Apolipoprotein E* (*APOE*) is the most established gene associated with the pathogenesis of late-onset Alzheimer’s disease (AD) [1]. Presence of an *APOE*-ε4 allele not only increases the risk of AD but also facilitates the rate of cognitive decline during the course of disease [2]. Pathogenic studies show that the *APOE*-ε4 allele is associated with reduction of APOE protein [3] that subsequently leads to several AD-relevant changes, including memory impairment, synaptogenesis dysfunction, tau hyperphosphorylation [4], and amyloid β (Aβ) plaque deposition [5], which together may hasten the progression of AD. Because inducible expression of APOE to facilitate Aβ clearance [6], reorganize synaptic disruption [7], and slow cognitive decline [8] may provide novel approaches to AD treatment, there is a critical need to dissect the effects of *APOE* on AD biomarkers. Resting-state functional magnetic resonance imaging (rs-fMRI) is one of the most important biomarkers of AD, since functional connectivity in the default mode network (DMN) closely tracks clinical deterioration and can be reliably measured in AD [9]. In rs-fMRI studies, changes in DMN connectivity due to the *APOE*-ε4 allele have not been determined, as one study showed no *APOE*-related differences in functional connectivity [10], a second study reported significantly lower DMN connectivity in *APOE*-ε4 allele carriers than in *APOE*-ε3 homozygotes [11], and a third study demonstrated increased DMN connectivity in *APOE*-ε4 allele carriers [12].

A second gene that might serve as a target gene for therapy is the *ATP-binding cassette*, *sub-family A*, *member 7* (*ABCA7*) that codes for the ATP-biding cassette transporter A7 [13]. *ABCA7* protein is abundantly expressed in the brain [13] and is heavily expressed in the hippocampus [14]*.* Similar to APOE, ABCA7 plays a critical role in Aβ generation and plaque formation. ABCA7 deficiency facilitates amyloidogenic processing by increasing the levels of β-secretase to cleave Aβ from amyloid precursor protein (APP) [15]. The presence of an rs3764650G allele in *ABCA7* has been associated with the development of neuritic plaque pathology [16], which has been attributed to ABCA7 deficiency [15]. Conversely, carrying the rs3764650T allele in *ABCA7* shows a protective effect against Aβ plaque formation in AD [17]. These data suggest that increasing the expression of ABCA7 may be another candidate therapeutic target that may reduce neuritic plaque burden and reorganize the fibrillar amyloid burden-mediated disruption of functional connectivity in the DMN [7].

Both *APOE*-ε4 allele [18] and APOE dysfunction [19] have pronounced and widely replicated effects on increased Aβ plaque deposition. Following this pattern, one would expect that the *APOE*-ε4 allele has a deleterious effect on memory [8] and DMN connectivity [7]. The marked lack of replicability of *APOE*-related alteration of the DMN in these studies [10–12] might be because they overlooked potential genetic interactions. There are two reasons for investigating the potential effects of *APOE-ABCA7* interactions on DMN alteration and its associated memory impairment in AD; one is that loss-of-function variants in both *APOE* and *ABCA7* drive Aβ plaque deposition [15, 19], and the other is because the *ABCA7* (rs3764650) and *APOE*-ε4 carrier status show interaction effects on memory [20].

There are at least three DMN subsystems of interest to AD pathology. One subsystem, termed the posterior DMN (pDMN), includes the posterior cingulate cortex (PCC), medial prefrontal cortex, and temporoparietal junction (TPJ), and is anchored by the PCC seed [21]. Another subsystem, termed the anterior DMN (aDMN), includes the dorsal medial prefrontal cortex (DMPFC), TPJ, lateral temporal cortex, and temporal pole, and is anchored by the DMPFC seed [22]. Still, another subsystem, termed either the medial temporal lobe (MTL) subsystem [23] or the ventral DMN (vDMN) [24], includes the posterior inferior parietal gyrus, posterior parahippocampal gyrus, hippocampus, and entorhinal cortex, and is anchored by the entorhinal seed [25].

Several studies have investigated the influence of the *APOE*-ε4 carrier status on the modulation of default mode subnetworks [24]. The effects of the genetic interaction on the functional connectivity in each DMN subsystem have not been elucidated. The pDMN is mainly involved in autobiographical memory, the vDMN in constructing memory-based mental scenes, and the aDMN in self-referential processing [23, 26]. We would expect that the genetic interaction affects subnetworks that are more directly involved with memory function. In the present study, we investigated the effect of the *APOE* and *ABCA7* (rs3764650) interaction on memory and functional connectivity in each DMN subsystem. We aimed to explore the contribution of these genetic variants to specific subnetworks and memory.

## Methods

### Inclusion and exclusion criteria

Two hundred and eighty-seven patients with typical AD were enrolled from the Department of Neurology of Chang Gung Memorial Hospital. The patients were included after consensus from a panel composed of neurologists, neuropsychologists, neuroradiologists, and experts in nuclear medicine [27]. Probable AD was diagnosed according to the Recommendations from the National Institute on Aging and the Alzheimer’s Association workgroup [28], with a clinical phenotype of early and significant memory impairment. All AD patients with dementia syndrome were under stable treatment with acetylcholine esterase inhibitors (AChEIs) from the time of diagnosis. Only the AD patients with mild to moderate dementia, having a Clinical Dementia Rating (CDR) score of 0.5, 1, or 2, in the status of stable blood pressure, and with medication on a stable dose were included. The exclusion criteria were a modified Hachinski ischemic score > 4 [29] and a negative amyloid scan by visual interpretations. The amyloid scans used (18) F-florbetapir positron-emission tomography imaging. The protocol was described in our previous studies [30]. Visual rating of a negative amyloid scan was based on the inspection of predominantly white matter tracer retention with no appreciable cortical gray matter (GM) retention above cerebellar GM levels. The nuclear medicine physicians were blinded to clinical information.

### Study design

This study was approved by the Institutional Review Committee on Human Research of Chang Gung Memorial Hospital, and all of the participants and their authorized caregivers provided written informed consent. Cognitive testing and all MRI were performed within a 4-week period.

### Genotyping

Genomic DNA was extracted from blood samples using a commercial kit (Qiagen, Gentra Puregene Blood Kit, USA), followed by genotyping for the G1527T SNP in the *ABCA7* gene using the polymerase chain reaction-restriction fragment length polymorphism method [17]. The *APOE* genotype was also determined [31]. Genotyping was conducted with the operator blinded to the clinical data. The patients were classified into two genotypic groups based on the *ABCA7* rs3764650T allele homozygous carriers (TT-carriers) and *ABCA7* rs3764650G allele carriers (G-allele-carriers). Those with one or two *APOE*-ε4 alleles were defined as *APOE*-ε4 carriers (ε4+ carriers) and the others as *APOE*-ε4 non-carriers (ε4 non-carriers). Among the patients, 86 ε4+ carriers were heterozygous (ε3/ε4), 10 ε4+ carriers were homozygous (ε4/ε4), 165 patients were homozygous for the ε3 allele (ε3/ε3), 21 patients had the ε2/ε3 genotype, and 5 patients had the ε2/ε4 genotype. The *χ*^2^ test was used to assess whether the allele frequencies agreed with expectation of the Hardy-Weinberg equilibrium (HWE). Statistical significance was set at *P* < 0.05.

### MRI acquisition and pre-processing

MRI was acquired on a GE Signa Excite 3.0 T scanner (GE Medical System, Milwaukee, WI). The protocols of the T1-weighted imaging scanning, structural MRI pre-processing, and rs-fMRI pre-processing steps, including slice time correction, realignment, segmentation, normalization into standard stereotactic Montreal Neurological Institute (MNI) spaces, spatial smoothing using a Gaussian Kernel of 6 mm, and resampling to 2 × 2 × 2 mm^3^, were described in our previous study [32]. In rs-fMRI pre-processing, to remove motion artifacts, variations in the average blood oxygen level-dependent signal from scan to scan should be less than 1%. In addition, framewise displacement should be less than 0.25 mm/TR. Simultaneously, images were detrended and filtered to a frequency between 0.008 and 0.09 Hz. Anatomical component-based noise correction methods, as implemented in the CONN toolbox (http://www.nitrc.org/projects/conn) [33], were used to regress out the head movement time series, white matter, and cerebrospinal fluid signals from each voxel.

### Neuropsychological assessments

Verbal memory was evaluated using the Chinese Version Verbal Learning Test (CVVLT) [34] that assessed the free recall of a number of items retrieved over four learning trials of a nine-word list after 30 s (CVVLT-30 sec) and after 10 min (CVVLT-10 min), and assessed the cued recall of a number of words retrieved with cued procedures over four learning trials (CVVLT-cued). CVVLT-30 sec and CVVLT-10 min tests were used to evaluate immediate and delayed free recalls. The subjects’ executive function was evaluated using the Trail Making Test B (TMB) [35]. The modified Rey-Osterrieth Complex Figure Copy test was used to assess visual-spatial abilities [36]. The CDR and Montreal Cognitive Assessment (MoCA) were used to assess the general intellectual function [37, 38]. The MoCA scores were converted to Mini-Mental State Examination (MMSE) scores subsequently [39].

### Statistical analysis

Clinical data were expressed as mean ± standard deviation. Independent *t* tests were used to compare continuous variables between the ε4+ versus ε4 non-carriers within the G-allele-carriers (G/ε4+ carriers versus G/ε4 non-carriers) and the TT-carriers (TT/ε4+ carriers versus TT/ε4 non-carriers). Interaction effects of *APOE* (ε4+ versus ε4 non-carriers) with *ABCA7* (TT- versus G-allele-carriers) on memory were also analyzed. We used a partial correlation to analyze the relationship between the neuroimaging data and memory scores. Stepwise regression analysis was used to determine the best predictors of memory performance. All statistical analyses were conducted using SPSS software (SPSS version 22 for Windows®, SPSS Inc., Chicago, IL). Statistical significance was set at *P* < 0.05.

For the ROI-based functional connectivity analysis, the pDMN was anchored by bilateral PCC seeds, two 10-mm-radius spheres centered on MNI coordinates (*x* = ± 4, *y* = − 45, *z* = 8) [21]. The PCC seeds have exhibited widespread connectivity with the other PCC/precuneus subregions and cortical areas [21]. The vDMN was anchored by bilateral entorhinal seeds, two 10-mm-radius spheres centered on MNI coordinates (*x* = ± 25, *y* = − 9, *z* = − 28) [25]. The entorhinal cortex was chosen as the seed region because it is linked with other subregions within the MTL [40] and with several neo-cortices [41]. The aDMN was anchored by bilateral DMPFC seeds, two 10-mm-radius spheres centered on MNI coordinates (*x* = ± 12, *y* = 51, *z* = 36) [22]. We used two-way analysis of variance (ANOVA) interaction analyses to determine the effect of *APOE-ABCA7* interactions on the functional connectivity of the bilateral DMPFC, entorhinal, and PCC seeds throughout the whole brain [33], using an uncorrected threshold of *P* < 0.001 at the peak level and cluster-level false discovery rates (FDR) corrected at *P* < 0.05. A seed reference time course was extracted by averaging the survived time course within each seed. ANOVA interaction analyses were carried out between the seed reference and whole brain in a voxel-wise manner. A Fisher’s *z*-transformation was applied to improve the normality of the correlation coefficients. The individual *z* value was entered into two-way ANOVA to determine brain regions significantly affected by interaction effects of *APOE* (ε4+ versus ε4 non-carriers) with *ABCA7* (TT- versus G-allele-carriers). The peak neural activity in each cluster of the t-maps for second-level analysis of ANOVA was extracted to correlate with the memory scores.

The correlation between neural activity in the DMN was also correlated with the memory scores in the CVVLT-30 sec, CVVLT-10 min, and CVVLT-cued tests using the multiple regression analysis in the CONN toolbox [33]. The peak cluster reference time course was obtained by averaging the survived time coursed within the peak cluster. Correlation analyses were carried out between peak cluster reference and memory scores. The correlation was computed using the CONN toolbox [33] with an uncorrected threshold of *P* < 0.001 at the peak level and a FDR corrected to *P* < 0.05 at the cluster level.

## Results

### Clinical and pathological characteristics

Two hundred eighty-seven patients with typical AD (127 TT-carriers and 160 G-allele-carriers) completed the study. There was no difference in the clinical and cognitive variables between the two genotypic groups (*P* > 0.05). The ε4+ carriers had significantly lower memory scores in the CVVLT-30 sec (*t* (286) = − 2.911; *P* = 0.004), CVVLT-10 min (*t* (286) = − 4.569; *P* < 0.001), and CVVLT-cued (*t* (286) = − 3.871; *P* < 0.001) tests than the ε4 non-carriers, but the two groups were not significantly different in any other clinical or cognitive variable (*P* > 0.05). The distributions of the homozygosity for rs3764650T allele and the *APOE*-ε4 carrier genotype conformed to the HWE, *χ*^2^ = 0.021 and *P* = 0.884 and *χ*^2^ = 0.077 and *P* = 0.782, respectively. Allele frequencies agreed with expectation of the HWE.

As the treatments such as sedatives and AChEIs may affect the functional connectivity in DMN [42], the number of patients that used these medications has been reported. In this study, all of our patients were under the stable treatment with AChEIs for at least 3 months. Moreover, in either G-allele-carriers or TT-carriers, ε4+ carriers and ε4 non-carriers did not have significant differences in proportion of patients who used sedatives (*P* > 0.05; Table 1).

**Table 1:** Demographic and clinical data of genotypic groups

	G-allele-carriers		*P* value	TT-carriers		*P* value
	ε4+ carriers	ε4 non-carriers		ε4+ carriers	ε4 non-carriers	
Sample size (*n*)	61	99		40	87	
Age (years)	72.8 ± 7.4	72.8 ± 7.7	0.945	72.3 ± 8.1	71.7 ± 9.1	0.689
Sex (female/male)	34/27	58/41	0.723	23/17	39/48	0.184
Education (years)	7.1 ± 4.6	7.0 ± 4.7	0.970	6.2 ± 5.4	8.0 ± 5.4	0.092
MMSE scores	18.6 ± 6.2	21.1 ± 7.1	0.023	20.0 ± 7.0	20.4 ± 7.2	0.735
Mean symptom duration (years)	2.5 ± 1.4	2.4 ± 1.2	0.686	2.4 ± 1.3	2.5 ± 1.0	0.676
Therapy affecting the default mode network						
Sedatives (*n*, %)	9, 14.8%	12, 12.1%	0.632	6, 15%	21, 24.1%	0.242
Memory scores						
CVVLT-30 sec	3.7 ± 2.6	5.0 ± 2.9	0.006	4.5 ± 3.0	5.1 ± 2.6	0.275
CVVLT-10 min	2.1 ± 2.9	4.6 ± 3.3	< 0.001	3.5 ± 3.5	4.3 ± 3.0	0.206
CVVLT-cued	3.0 ± 2.9	5.0 ± 2.8	< 0.001	4.1 ± 3.0	4.6 ± 2.8	0.388
Visuospatial function: mROCF-copy	13.8 ± 5.4	13.8 ± 5.2	0.950	13.4 ± 5.5	13.8 ± 5.2	0.675
Executive function scores: TMB (seconds)	105.2 ± 30.2	94.9 ± 35.1	0.061	95.1 ± 36.2	96.1 ± 32.3	0.868

Data are presented as mean ± standard deviation; *P* values denote significant differences between ε4+ and ε4− carriers in independent *t* tests for continuous variables and *χ*^2^ tests for dichotomous variables. *CVVLT* Chinese version of the Verbal Learning Test, *CVVLT-30 sec* recall after a 30-s interval, *CVVLT-10 min* recall after a 10-min delay, *CVVLT-cued* recall after a cue, *ε4+ carriers APOE*-ε4 carriers, *ε4 non-carriers APOE*-ε4 non-carriers, *G-allele-carriers ABCA7* rs3764650G allele carriers, *MMSE* Mini-Mental State Examination, *mROCF-copy* modified Rey-Osterrieth Complex Figure Copy test, *TMB* Trail Making Test B, *TT-carriers ABCA7* rs3764650T homozygous carriers

Since evidences have indicated impacts of age, *APOE*, and sex dimorphism on the risk of AD [43], we further analyzed the interaction effects of *APOE*-ε4 carriage with sex differences and with age of onset, in which subjects were dichotomized into early onset (before 65 years) or late onset disease (65 years and above) [44], on memory performances. In this study, we did not observe the interaction effects of *APOE*-ε4 carriage with sex differences (F3, 283 = 0.430; *P* = 0.512) or with age of onset (F3, 283 = 1.029; *P* = 0.311) on memory performances.

### Genotypic interaction effects on memory

In G-allele-carriers, the ε4+ carriers had significantly lower memory scores in the CVVLT-30 sec (*t* (159) = − 2.780; *P* = 0.006), CVVLT-10 min (*t* (159) = − 4.879; *P* < 0.001), and CVVLT-cued (*t* (159) = − 4.337; *P* < 0.001) tests, and lower MMSE scores (*t* (159) = − 2.298; *P* = 0.023) than ε4 non-carriers; the two groups did not have any other significant difference in clinical and cognitive variables (*P* > 0.05; Table 1).

In TT-carriers, the ε4+ carriers and ε4 non-carriers did not have significantly different clinical or cognitive variables (*P* > 0.05; Table 1), suggesting that homozygosity for *ABCA7* rs3764650T allele affected the difference between the ε4+ and ε4 non-carriers. Table 2 shows the effect of *APOE-ABCA7* interactions on scores in the CVVLT-10 min and CVVLT-cued tests (*P* < 0.05; Additional file 1: Figure S1). Meanwhile, *APOE*-ε4 carriage showed a leading effect on memory impairment (*P* < 0.05; Table 2).

**Table 2:** Significant two-way interaction for each dependent variable

Memory function scores	Main effects	F3, 283	*P* value
CVVLT-30 sec score	*APOE-*ε4 carrier genotype	6.953	0.009
	*ABCA7* (rs3764650)	1.985	0.160
	*APOE-*ε4 carrier genotype *× ABCA7* (rs3764650)	0.928	0.336
CVVLT-10 min score	*APOE-*ε4 carrier genotype	17.004	< 0.001
	*ABCA7* (rs3764650)	2241	0.135
	*APOE-*ε4 carrier genotype *× ABCA7* (rs3764650)	4.755	0.030
CVVLT-cued score	*APOE-*ε4 carrier genotype	12.039	0.001
	*ABCA7* (rs3764650)	0.927	0.337
	*APOE-*ε4 carrier genotype *× ABCA7* (rs3764650)	4.611	0.033

*CVVLT* Chinese version of the Verbal Learning Test, *CVVLT-10 min* recall after a 10-min delay, *CVVLT-cued* recall after a cue)

Disease severity was further controlled in the interactional analysis by grouping patients into patients with CDR = 0.5 or CDR = 0.5 or 1 (Additional file 2: Table S1), and there was still a significant *APOE-ABCA7* interaction effect on scores in CVVLT-10 min (*P* < 0.05).

### Genetic interaction showing dissociable effects on functional connectivity in the ventral DMN and posterior DMN

**Fig. 1:**
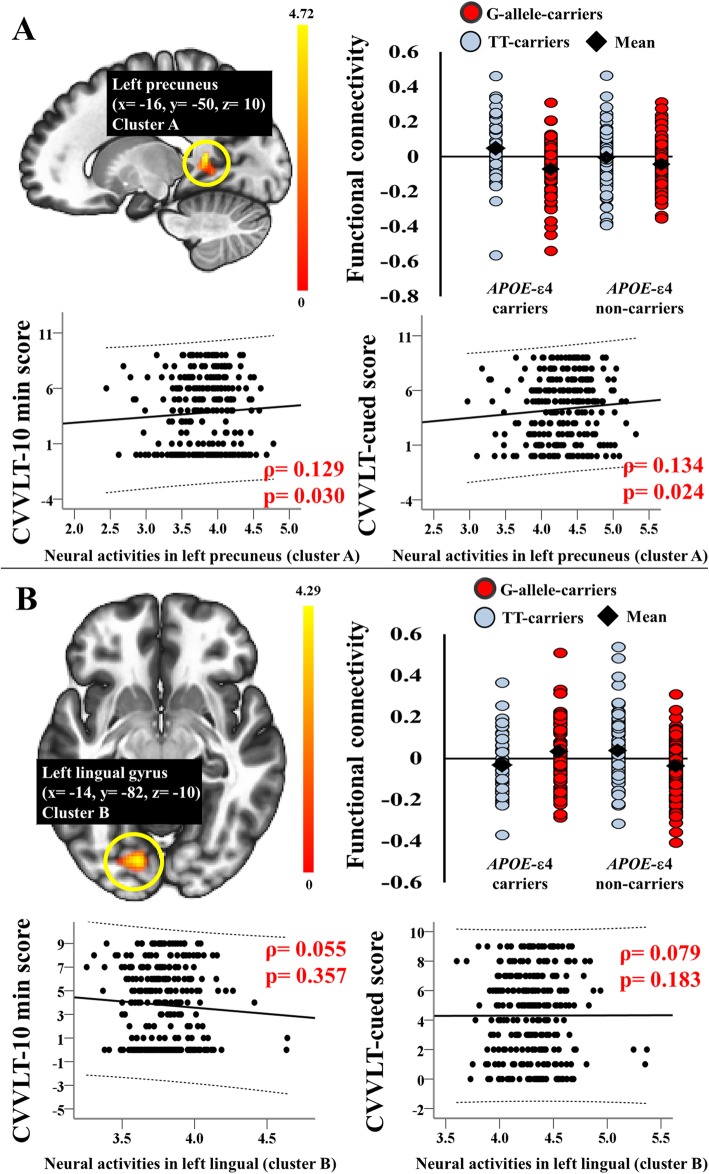
An *APOE-ABCA7* (rs3764650) interaction effect on functional connectivity in the default mode network anchored by the right entorhinal seed (**a**) and left posterior cingulate cortical seed (**b**), and the correlation between the genetic interaction-associated neural activity and memory scores in the CVVLT-10 min and CVVLT-cued tests. Residuals are plotted to determine the correlation between each variable. The 95% confidence interval is the area enclosed by the dashed curves. (*x*, *y*, *z*), coordinates on Montreal Neurological Institute template brain; CVVLT, Chinese version of the Verbal Learning Test; G-allele-carriers, *ABCA7* rs3764650G allele carriers; TT-carriers, *ABCA7* rs3764650T homozygous carriers

We used two-way ANOVA interaction analyses to determine the effect of the *APOE-ABCA7* interactions on functional connectivity in the bilateral DMPFC and entorhinal and PCC seeds with the voxels throughout the whole brain. Figure 1 shows the significant interaction effect of *APOE* with *ABCA7* on the functional connectivity between the right entorhinal seed and left precuneus (uncorrected *P* at peak level = 0.00002; Additional file 3: Table S2) and on functional connectivity between the left PCC seed and the left lingual gyrus (uncorrected *P* at peak level = 0.00001; Additional file 3: Table S2). Since there were *APOE-ABCA7* interaction effects on scores in the CVVLT-10 min and CVVLT-cued tests, the peak neural activity in each cluster that was associated with the effect *APOE-ABCA7* interactions was extracted to correlate with the memory scores in the CVVLT-10 min and CVVLT-cued tests.

In the DMN anchored by the right entorhinal seed, the peak neural activity of the cluster that was significantly associated with *APOE*-*ABCA7* interaction effects were correlated with memory scores in the CVVLT-10 min and CVVLT-cued tests (*P* < 0.05; Fig. 1). However, in the DMN anchored by the left PCC seed, the peak neural activity of the cluster that was significantly associated with *APOE*-*ABCA7* interaction effects were not correlated with any memory scores (*P* > 0.05; Fig. 1).

### Functional connectivity in the DMN and its clinical significance

Scores in the CVVLT-30 sec test were correlated with neural activity in the insula in the pDMN anchored by the bilateral PCC seeds, the orbitofrontal gyri and lingual gyri in the vDMN anchored by the bilateral entorhinal seeds, and the medial frontal gyri in the aDMN anchored by the bilateral DMPFC seeds (peak-level uncorrected *P* < 0.001; Additional file 4: Table S3).

Scores in the CVVLT-10 min test were correlated with neural activity in the left insula in the pDMN anchored by the left PCC seed, the left anterior cingulate cortex in the vDMN anchored by the left entorhinal seed, the left lingual and middle orbitofrontal gyrus in the vDMN anchored by the right entorhinal seed, and the inferior frontal gyri in the aDMN anchored by the bilateral DMPFC seeds (peak-level uncorrected *P* < 0.001; Additional file 4: Table S3).

Scores in the CVVLT-cued test were correlated with neural activity in the insula in the pDMN anchored by bilateral PCC seeds, the right PCC in the vDMN anchored by the right entorhinal seed, the bilateral orbitofrontal gyri in the vDMN anchored by the bilateral entorhinal seeds, and the frontal gyri in the aDMN anchored by the bilateral DMPFC seeds (peak-level uncorrected *P* < 0.001; Additional file 4: Table S3).

With a FDR-corrected *P* < 0.05 at the peak level and cluster level, scores in CVVLT-cued were correlated with neural activity in the frontal areas in the aDMN anchored by the bilateral DMPFC seeds. Score in CVVLT-10 min was correlated with neural activity in left orbitofrontal and left inferior temporal gyri in vDMN anchored by right entorhinal seed (FDR-corrected *P* < 0.05; Additional file 5: Table S4), in which there was a significant interaction effect of *APOE* with *ABCA7* on the functional connectivity between the right entorhinal seed and left precuneus (uncorrected *P* at peak level = 0.00002; Additional file 3: Table S2).

## Discussion

### Main findings

There were three main findings in the current study. First, we demonstrated the genetic interaction effect of *APOE* with *ABCA7* (rs3764650) on memory scores. Second, although functional connectivity in the aDMN, pDMN, and vDMN was shown to be a biologically relevant signal, the present study only identified a significant effect of *APOE*-*ABCA7* interactions on neural activities in the DMN anchored by the right entorhinal and left PCC seeds. Third, in the vDMN, neural activity that was significantly associated with *APOE*-*ABCA7* interaction effects was positively correlated with memory scores.

### Interaction effects of *APOE* with *ABCA7* on functional connectivity in the DMN

Since functional connectivity in the DMN tracks clinical deterioration in AD [9], altered functional connectivity in the DMN has become one of the most important biomarkers of AD. However, the influence of genetic effects on changes to DMN connectivity has not been well estimated. In the present study, we demonstrated that genetic interaction might affect DMN connectivity in patients with AD.

The effects of *APOE-ABCA7* interaction on functional connectivity in the DMN might operate through both Aβ-mediated and non-Aβ-mediated metabolism in the brain. As a protein of the ATP-binding cassette transporter [13], ABCA7 expression appears to modulate the intracellular cholesterol metabolism pathway within microglia [45]. By acting as neuroprotective and neurotrophic cells, microglia are known to be involved in rescuing neurons, modifying neural synapses, and regulating neuronal transmission within the pathological brain [46]. The impact of *ABCA7* polymorphisms on functional connectivity in the DMN might be mediated by modulating microglia function. The effect of ABCA7 deficiency-associated microglia dysfunction on neural connectivity and the influence of APOE dysfunction*-*dependent accumulation of phosphorylated tau [4] on network integrity [47] are potential non-Aβ-mediated bases for the *APOE-ABCA7* interaction effect on functional connectivity in the DMN. In addition, ABCA7 expression has been shown to share 54% sequence identity to ABCA1 protein [48], which is involved with lipidation of APOE and is suggested to counteract the status of *APOE*-ε4 carriage-related hypolipidation of APOE [49]. Regarding the protein-protein interaction of ABCA7 with APOE, ABCA7 expression might be associated with facilitating the lipidation of APOE, thereby reversing pathological effect of hypolipidated APOE on synaptic impairment [50].

In addition to non-Aβ-mediated metabolism, Aβ metabolism in the brain could also cause a genetic interaction effect that reduces functional connectivity in the DMN. APOE expression appears to facilitate Aβ clearance and modulate cognitive and synaptic dysfunction [6, 7]. ABCA7 deficiency increases the cleavage of Aβ from APP by β-secretase [15]. *ABCA7* rs3764650G allele carriage is a loss-of-function variant [3, 15, 16]. Because substantial Aβ burden is associated with disruptions to functional connectivity [7], the changes in functional connectivity in *ABCA7* rs3764650G allele carriers might be associated with amyloidogenic processing facilitated by ABCA7 dysfunction. ABCA7 expression may be able to modulate the deleterious effect of *APOE*-ε4 carriage on synaptic connectivity [11] and memory performance [20]. Therefore, ABCA7 expression may be a candidate therapeutic target that could reduce neuritic plaque burden and fibrillar amyloid burden-mediated disruption of functional connectivity in the DMN.

The present study demonstrated the effect of *APOE*-*ABCA7* interactions on functional connectivity in the vDMN, and peak neural activity of the cluster was used as a biologically relevant signal. The vDMN activation is involved with constructing mental scenes based on memory [23, 24]. We suspected that increasing ABCA7 expression may benefit memory processing through the vDMN in *APOE*-ε4 carriers.

Regarding the effect of *APOE*-*ABCA7* interactions on functional connectivity in the vDMN, previous studies have reported that the hippocampal atrophy is associated with *ABCA7* rs3764650G allele [51] and *APOE*-ε4 carriage [49]. However, the effect of ABCA7 or APOE deficiency on PCC and DMPFC was less pronounced [49, 52]. The marked influence of *APOE*-*ABCA7* interactions on vDMN may be associated with the relationship between MTL atrophy and the genetic interaction effect.

In the present study, memory scores were positively correlated with neural activity in all the DMN subnetworks, including aDMN, pDMN, and vDMN. In addition, *APOE*-*ABCA7* interaction was associated with neural activities in both the vDMN and pDMN; however, only neural activity in the vDMN that showed an association with the effect of *APOE*-*ABCA7* interaction was correlated with memory. Using a restrictive analysis with FDR-corrected *P* < 0.05 at both peak and cluster levels, delayed free recalls were only correlated with functional connectivity in vDMN. This data suggests that the vDMN is preferentially engaged in the relationship between the genetic effects and memory impairment in AD.

### Interaction effects of *APOE* with *ABCA7* on memory

Carrying the *ABCA7* rs3764650T allele is associated with an increased expression of ABCA7 [17]. In the present study, TT-carriers tended to have worse memory than G-allele-carriers within *APOE*-ε4 non-carriers, but better memory than G-allele-carriers within *APOE*-ε4 carriers. In this regard, we suggest that *APOE*-ε4 carrier status is implicated in the pathogenesis of expression of ABCA7-associated clinical and pathological heterogeneity.

Regarding the association between *APOE*-ε4 and memory, it has been shown that *APOE*-ε4 has a deleterious effect on memory [2]. We also observed that *APOE*-ε4 carriage showed a leading pathological effect on memory impairment. Moreover, the deleterious influence of the presence of *APOE*-ε4 allele seemed to be modulated by the carriage of homozygosity for *ABCA7* rs3764650T allele. In TT-carriers in this study, we found that memory scores of ε4+ carriers were not worse than those of ε4 non-carriers, suggesting a protective effect of homozygosity for *ABCA7* rs3764650T allele against memory impairment in AD patients that carried *APOE*-ε4 allele even though the TT-carriers who carried *APOE*-ε4 allele had a trend of lower educational level than those who were *APOE*-ε4 non-carriers.

The effect of *APOE*-*ABCA7* interactions on memory performance is also shown in one previous study [20]. However, it demonstrates that the presence of the *ABCA7* rs3764650G allele genotype protects against the deleterious effect of the *APOE*-ε4 allele on memory performance [20]. Interestingly, our findings of the *APOE*-*ABCA7* interactions on functional connectivity in the DMN showed that the *ABCA7* rs3764650G allele was associated with higher functional connectivity within the pDMN in ε4+ carriers. However, we failed to show a direct relationship between neural activities in the pDMN that showed an association with the effect of *APOE*-*ABCA7* interaction was correlated with any memory scores. Conversely, we showed that homozygosity for *ABCA7* rs3764650T allele shows a protective effect against memory impairment in AD patients that carry *APOE*-ε4, and TT-carriers had higher functional connectivity within the vDMN in ε4+ carriers. Not only *APOE*-*ABCA7* interaction-associated functional connectivity in the vDMN rather than in the pDMN was associated with memory score, but also we demonstrated that delayed free recalls were only correlated with functional connectivity in vDMN in the restrictive analysis. Taken together, the effect of genetic interaction on the neural activity in the vDMN might affect memory performance in the symptomatic stage of AD. Meanwhile, as the pDMN has been shown to start disengaging in the earliest phase of AD [9], the genetic interaction effect on neural activity in the pDMN may affect memory performance in the pre-symptomatic stage of AD [20].

### The relationship between memory scores and DMN

Reduced functional connectivity in the DMN has been associated with memory dysfunction in the early stage of AD [53]. To further demonstrate the direct relationship between DMN and out-of-scanner memory performances in AD, we correlated the memory function score with functional connectivity in the posterior, anterior, and ventral DMN (pDMN, aDMN, and vDMN). Functional connectivity strength varies according to time and frequency in the resting state, but it is reported to provide an average representation across the entire time series [54]. This measure of functional connectivity has been correlated with cognitive performance and is considered to be a clinically relevant signal [55]. Verbal memory performances, assessed using California Verbal Learning Test, have been associated with neural activity in the DMN in cognitively normal aging [56]. In agreement with the previous study, the present study demonstrated the relationship between activity in the DMN and out-of-scanner memory performance, emphasizing the role of the DMN in AD-associated memory impairment.

Before suggesting memory attributes for distinct subnetworks, we first discuss the convergence among the aDMN, pDMN, and vDMN. In the present study, the scores in immediate free recalls, delayed free recalls, and cued recalls were associated with neural activity in the three subnetworks. Moreover, we observed dissociable patterns of association between memory and neural activity in the aDMN, pDMN, and vDMN. In the vDMN, all memory scores were associated with the functional connectivity of bilateral entorhinal seeds with orbitofrontal, temporal, and cingulate gyri, typical regions involved in the vDMN [57]. Similarly, in the aDMN, most of the memory scores were associated with functional connectivity of bilateral DMPFC seeds with middle and inferior frontal gyri, typical regions involved in aDMN [22]. However, in the pDMN, all memory scores were associated with functional connectivity between PCC seeds and left insula. The pathogenesis underlying the neural activity of insula in memory may be associated with non-specific memory network including the hippocampus, precuneus, and insula [58]. Considering the number of comparisons, more restrictive analysis with FDR-corrected *P* < 0.05 at peak level showed that cued recalls were especially associated with inferior or middle frontal gyri in aDMN anchored by bilateral DMPFC seeds, and neural activity in vDMN anchored by right entorhinal seed was associated with delayed free recalls. Meanwhile, there were genetic interaction effects on functional connectivity in vDMN anchored by right entorhinal seed and delayed free recalls. Interestingly, this study showed that *APOE-ABCA7* interaction-associated functional connectivity between the right entorhinal seed and left precuneus was correlated with verbal memory impairment. A previous study shows that disruption of hippocampus-precuneus functional connectivity plays a critical role in AD [59]. A decrease in the structural association between the right entorhinal cortex and medial prefrontal cortex is suggested to give rise to the progressive verbal memory impairment in AD [25]. Furthermore, the magnitude and extent of regions showing reduced connectivity to the right hippocampus is greater than that to the left hippocampus in AD patients as compared with elderly controls, suggesting that rightward hippocampal connectivity disruption may have a greater impact on the pathogenesis of AD [60]. Overall, our results are generally concordant with studies showing the importance of functional connectivity between the entorhinal cortex and precuneus in the clinical and pathological changes in AD, and the DMN anchored by the right entorhinal seed might be sensitive for studying pathophysiological changes in AD.

The effect of genetic interaction on the DMN suggests that genetic-biological systems may impact disease presentation and therapy. Cholinergic esterase inhibitors are among the only treatments available for AD because of their cholinergic effects on functional connectivity in the DMN [61]. Clarifying the effect of *APOE-ABCA7* interactions on the DMN and memory might be helpful to refine therapeutic management by increasing APOE and ABCA7 expression.

### Limitations

First, the gene-gene interaction effects derived from only two single nucleotide polymorphisms may not be able to fully explain the pathological changes in AD. Second, longitudinal data and genetic interaction effects on AD pathology (such as amyloid scan) will be needed to further investigate disease mechanisms of AD. Third, further studies will be needed to investigate the relationship between ABCA7 expression level and DMN and to uncover the modulation effect of *ABCA7* polymorphism on the DMN. Fourth, some of our patients received sedatives, which might affect functional connectivity in DMN to some degree, and further studies will be needed to evaluate the influence of these medications.

## Conclusions

The present study demonstrated that the *APOE*-*ABCA7* interaction affected memory and synaptic connectivity in the vDMN. The effect of the *APOE*-*ABCA7* interaction on the vDMN might influence clinical performance. Clarifying the effect of *APOE-ABCA7* interactions on the DMN and memory might be helpful to refine therapeutic management by increasing APOE and ABCA7 protein expression.

## Supplementary information

**Additional file 1 : Figure S1.** APOE-ABCA7 (rs3764650) interaction effects on scores in the CVVLT-10 min and CVVLT-cued tests.

**Additional file 2 : Table S1.** Significant two-way interaction for each dependent variable.

**Additional file 3 : Table S2.** Seed-to-voxel analysis reveals brain regions with significant effects of APOE-ABCA7 (rs3764650) interactions on functional connectivity in brain networks.

**Additional file 4 : Table S3.** Correlations of activity in brain regions in networks anchored by each seed of default mode network with each memory function score

**Additional file 5 : Table S4.** Correlations of activity in brain regions in networks anchored by each seed of default mode network with each memory function score.

## Data Availability

The datasets used and/or analyzed during the current study are available from the corresponding author on reasonable request.

## Abbreviations

Aβ: Amyloid β
AChEIs: Acetylcholine esterase inhibitors
AD: Alzheimer’s disease
ANOVA: Analysis of variance
CDR: Clinical Dementia Rating
CVVLT: Chinese Version Verbal Learning Test
DMN: Default mode network (aDMN, anterior DMN; pDMN, posterior DMN; ventral DMN, vDMN)
DMPFC: Dorsal medial prefrontal cortex
ε4+ carriers: *APOE*-ε4 carriers
ε4 non-carriers: *APOE*-ε4 non-carriers
FDR: False discovery rates
G-allele-carriers: *ABCA7-*rs3764650-G-allele genotype
GM: Gray matter
HWE: Hardy-Weinberg equilibrium
MMSE: Mini-Mental State Examination
MNI: Montreal Neurological Institute
MoCA: Montreal Cognitive Assessment
MRI: Magnetic resonance imaging
MTL: Medial temporal lobe
NCs: Normal controls
PCC: Posterior cingulate cortex
SNPs: Single nucleotide polymorphisms
TPJ: Temporoparietal junction
TT-carriers: *ABCA7-*rs3764650-TT genotype
