# Supplementary material for: ABCA7 polymorphisms correlate with memory impairment and default mode network in patients with APOEε4-associated Alzheimer’s disease
Source: Alzheimers Res Ther. 2019 Dec 12;11:103. doi: 10.1186/s13195-019-0563-3 (PMC6909474; doi:10.1186/s13195-019-0563-3)
Supplement: Supplementary file 1 — Additional file 1 : Figure S1. APOE-ABCA7 (rs3764650) interaction effects on scores in the CVVLT-10 min and CVVLT-cued tests. [file 13195_2019_563_MOESM1_ESM.docx]

**Title**

*ABCA7* Polymorphisms Correlate with Memory Impairment and Default Mode Network in Patients with *APOE*ε4 Associated Alzheimer’s Disease

**Journal name**

Alzheimer’s research & therapy

**Author names**

Ya-Ting Chang*^1^ MD, PhD; Shih-Wei Hsu^2^, MD; Shu-Hua Huang^3^ MD; Chi-Wei Huang^1^ MD, PhD; Wen-Neng Chang^1^ MD; Chia-Yi Lien^1^ MD; Jun-Jun Lee^1^ MD; Chen-Chang Lee^2^ PhD; Chiung-Chih Chang*^1^ MD, PhD

^1^Department of Neurology, Institute of translational research in biomedicine, Kaohsiung Chang Gung Memorial Hospital, Chang Gung University College of Medicine, Kaohsiung 83301, Taiwan

^2^Department of Radiology, Kaohsiung Chang Gung Memorial Hospital, Chang Gung University College of Medicine, Kaohsiung, Taiwan

^3^Department of Nuclear Medicine, Kaohsiung Chang Gung Memorial Hospital, Chang Gung University College of Medicine, Kaohsiung, Taiwan

*Ya-Ting Chang and Chiung-Chih Chang are co‐corresponding authors

Submission Type: Article

**Figure S1**

**
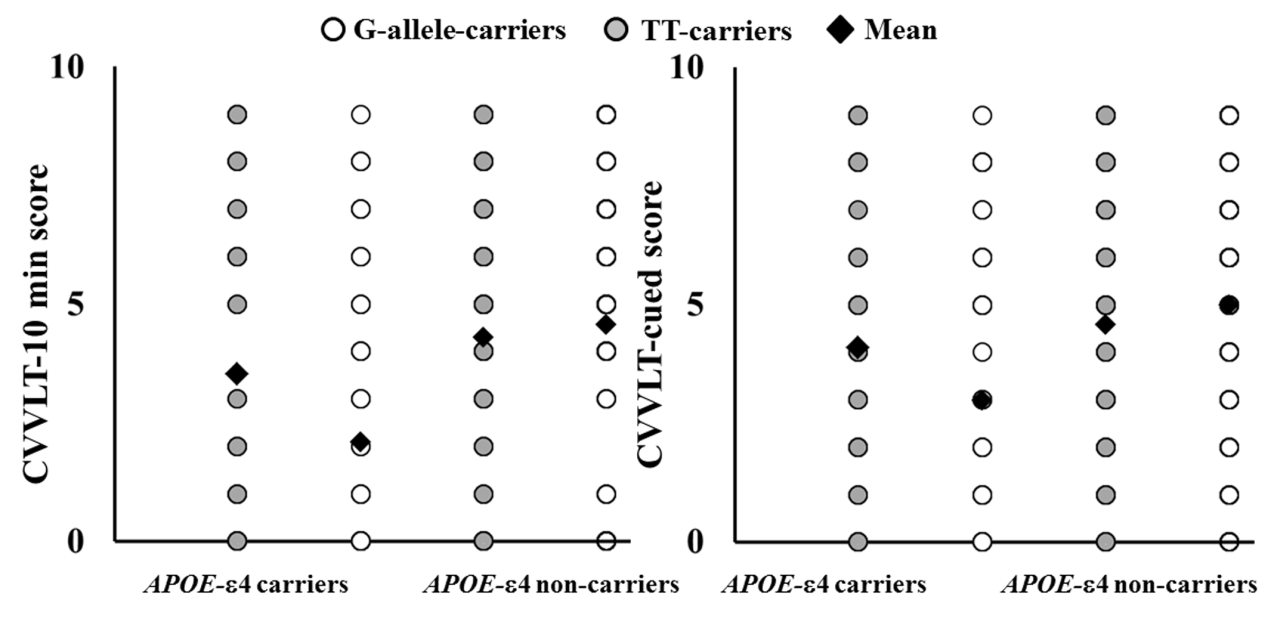
**

**Figure S1** *APOE-ABCA7* (rs3764650) interaction effects on scores in the CVVLT-10 min and CVVLT-cued tests. CVVLT, Chinese version of the Verbal Learning Test; G-allele-carriers, *ABCA7* rs3764650G allele carriers; TT-carriers, *ABCA7* rs3764650T homozygous carriers.
