# Supplementary material for: ABCA7 polymorphisms correlate with memory impairment and default mode network in patients with APOEε4-associated Alzheimer’s disease
Source: Alzheimers Res Ther. 2019 Dec 12;11:103. doi: 10.1186/s13195-019-0563-3 (PMC6909474; doi:10.1186/s13195-019-0563-3)
Supplement: Supplementary file 2 — Additional file 2 : Table S1. Significant two-way interaction for each dependent variable. [file 13195_2019_563_MOESM2_ESM.docx]

**Title**

*ABCA7* Polymorphisms Correlate with Memory Impairment and Default Mode Network in Patients with *APOE*ε4 Associated Alzheimer’s Disease

**Journal name**

Alzheimer’s research & therapy

**Author names**

Ya-Ting Chang*^1^ MD, PhD; Shih-Wei Hsu^2^, MD; Shu-Hua Huang^3^ MD; Chi-Wei Huang^1^ MD, PhD; Wen-Neng Chang^1^ MD; Chia-Yi Lien^1^ MD; Jun-Jun Lee^1^ MD; Chen-Chang Lee^2^ PhD; Chiung-Chih Chang*^1^ MD, PhD

^1^Department of Neurology, Institute of translational research in biomedicine, Kaohsiung Chang Gung Memorial Hospital, Chang Gung University College of Medicine, Kaohsiung 83301, Taiwan

^2^Department of Radiology, Kaohsiung Chang Gung Memorial Hospital, Chang Gung University College of Medicine, Kaohsiung, Taiwan

^3^Department of Nuclear Medicine, Kaohsiung Chang Gung Memorial Hospital, Chang Gung University College of Medicine, Kaohsiung, Taiwan

*Ya-Ting Chang and Chiung-Chih Chang are co‐corresponding authors

Submission Type: Article

**Table S1** Significant two-way interaction for each dependent variable

| **Only patients with CDR = 0.5** | **Main effects** | | **F3, 194** | **p value** |
| --- | --- | --- | --- | --- |
| CVVLT-10 min score | | *APOE-*ε4 carrier genotype | 13.221 | <0.001 |
|  | | *ABCA7* (rs3764650) | 1.631 | 0.203 |
| *APOE-*ε4 carrier genotype x *ABCA7* (rs3764650) | | | 4.510 | 0.035 |
| **Only patients with CDR = 0.5 or 1** | | **Main effects** | **F3, 270** | **p value** |
| CVVLT-10 min score | | *APOE-*ε4 carrier genotype | 16.939 | <0.001 |
|  | | *ABCA7* (rs3764650) | 3.299 | 0.070 |
| *APOE-*ε4 carrier genotype x *ABCA7* (rs3764650) | | | 4.032 | 0.046 |

CDR, Clinical Dementia Rating; CVVLT, Chinese version of the Verbal Learning Test (CVVLT-10 min, recall after a 10-minute delay).
