# Supplementary material for: ABCA7 polymorphisms correlate with memory impairment and default mode network in patients with APOEε4-associated Alzheimer’s disease
Source: Alzheimers Res Ther. 2019 Dec 12;11:103. doi: 10.1186/s13195-019-0563-3 (PMC6909474; doi:10.1186/s13195-019-0563-3)
Supplement: Supplementary file 3 — Additional file 3 : Table S2. Seed-to-voxel analysis reveals brain regions with significant effects of APOE-ABCA7 (rs3764650) interactions on functional connectivity in brain networks. [file 13195_2019_563_MOESM3_ESM.docx]

**Title**

*ABCA7* Polymorphisms Correlate with Memory Impairment and Default Mode Network in Patients with *APOE*ε4 Associated Alzheimer’s Disease

**Journal name**

Alzheimer’s research & therapy

**Author names**

Ya-Ting Chang*^1^ MD, PhD; Shih-Wei Hsu^2^, MD; Shu-Hua Huang^3^ MD; Chi-Wei Huang^1^ MD, PhD; Wen-Neng Chang^1^ MD; Chia-Yi Lien^1^ MD; Jun-Jun Lee^1^ MD; Chen-Chang Lee^2^ PhD; Chiung-Chih Chang*^1^ MD, PhD

^1^Department of Neurology, Institute of translational research in biomedicine, Kaohsiung Chang Gung Memorial Hospital, Chang Gung University College of Medicine, Kaohsiung 83301, Taiwan

^2^Department of Radiology, Kaohsiung Chang Gung Memorial Hospital, Chang Gung University College of Medicine, Kaohsiung, Taiwan

^3^Department of Nuclear Medicine, Kaohsiung Chang Gung Memorial Hospital, Chang Gung University College of Medicine, Kaohsiung, Taiwan

*Ya-Ting Chang and Chiung-Chih Chang are co‐corresponding authors

Submission Type: Article

**Table S2** Seed-to-voxel analysis reveals brain regions with significant effects of *APOE-ABCA7* (rs3764650) interactions on functional connectivity in brain networks

| **Seed** | | **Cluster** | **MNI**  **(x, y, z)** | **Cluster size** | **T** | **p-FDR**  **of size** |
| --- | --- | --- | --- | --- | --- | --- |
| DMPFC | Right | No peak cluster |  |  |  |  |
|  | Left | No peak cluster |  |  |  |  |
| PCC | Right | No peak cluster |  |  |  |  |
|  | Left | Left lingual | -14, -82, -10 | 456 | 4.15 | 0.004 |
| Entorhinal | Right | Left precuneus | -16, -50, 10 | 472 | 4.91 | 0.006 |
|  |  | Left superior parietal gyrus | -32, -66, 48 | 662 | 3.92 | 0.001 |
|  | Left | No peak cluster |  |  |  |  |

T maxima, and contiguous voxels of cluster size are shown. The significance clusters are detected with thresholds of FDR-corrected P < 0.05 at the cluster-level and uncorrected P < 0.001 at the peak-level. DMPFC, dorsal medial prefrontal cortex; FDR, false discovery rate; MNI (x, y, z), local maxima coordinates on Montreal Neurological Institute template brain; PCC, posterior cingulate cortex.
