# Supplementary material for: ABCA7 polymorphisms correlate with memory impairment and default mode network in patients with APOEε4-associated Alzheimer’s disease
Source: Alzheimers Res Ther. 2019 Dec 12;11:103. doi: 10.1186/s13195-019-0563-3 (PMC6909474; doi:10.1186/s13195-019-0563-3)
Supplement: Supplementary file 4 — Additional file 4 : Table S3. Correlations of activity in brain regions in networks anchored by each seed of default mode network with each memory function score [file 13195_2019_563_MOESM4_ESM.docx]

**Title**

*ABCA7* Polymorphisms Correlate with Memory Impairment and Default Mode Network in Patients with *APOE*ε4 Associated Alzheimer’s Disease

**Journal name**

Alzheimer’s research & therapy

**Author names**

Ya-Ting Chang*^1^ MD, PhD; Shih-Wei Hsu^2^, MD; Shu-Hua Huang^3^ MD; Chi-Wei Huang^1^ MD, PhD; Wen-Neng Chang^1^ MD; Chia-Yi Lien^1^ MD; Jun-Jun Lee^1^ MD; Chen-Chang Lee^2^ PhD; Chiung-Chih Chang*^1^ MD, PhD

^1^Department of Neurology, Institute of translational research in biomedicine, Kaohsiung Chang Gung Memorial Hospital, Chang Gung University College of Medicine, Kaohsiung 83301, Taiwan

^2^Department of Radiology, Kaohsiung Chang Gung Memorial Hospital, Chang Gung University College of Medicine, Kaohsiung, Taiwan

^3^Department of Nuclear Medicine, Kaohsiung Chang Gung Memorial Hospital, Chang Gung University College of Medicine, Kaohsiung, Taiwan

*Ya-Ting Chang and Chiung-Chih Chang are co‐corresponding authors

Submission Type: Article

**Table S3** Correlations of activity in brain regions in networks anchored by each seed of default mode network with each memory function score

| **Seed**  **Memory score** | **Cluster** | **MNI**  **(x, y, z)** | **Cluster size** | **p-FDR**  **of size** | **T** |
| --- | --- | --- | --- | --- | --- |
| **Left DMPFC seed** |  |  |  |  |  |
| CVVLT-30 sec | Right superior medial frontal gyrus | 4, 60, 6 | 6456 | <0.001 | 5.66 |
| CVVLT-10 min | Right inferior frontal gyrus | 54, 24, 22 | 6046 | <0.001 | 5.76 |
| CVVLT-cued | Right inferior frontal gyrus | 54, 22, 22 | 9042 | <0.001 | 6.25 |
| **Right DMPFC seed** |  |  |  |  |  |
| CVVLT-30 sec | Left medial frontal gyrus | -8, 62, -4 | 2212 | <0.001 | 4.10 |
|  | Left middle frontal gyrus | -40, 22, 42 | 875 | <0.001 | 4.54 |
|  | Right middle frontal gyrus | 48, 14, 44 | 395 | 0.009 | 3.96 |
| CVVLT-10 min | Left middle frontal gyrus | -32, 42, 36 | 1813 | <0.001 | 5.19 |
|  | Right inferior frontal gyrus | 56, 18, 18 | 622 | 0.001 | 4.09 |
| CVVLT-cued | Left middle frontal gyrus | -42, 28, 40 | 1763 | <0.001 | 4.94 |
|  | Right middle frontal gyrus | 48, 16, 40 | 1330 | <0.001 | 4.66 |
|  | Left superior medial frontal gyrus | -12, 70, 0 | 940 | <0.001 | 3.85 |
| **Left entorhinal seed** |  |  |  |  |  |
| CVVLT-30 sec | Right middle orbitofrontal gyrus | 28, 48, -14 | 3819 | <0.001 | 4.94 |
|  | Right lingual gyrus | 18, -86, -14 | 1402 | <0.001 | 4.92 |
|  | Right fusiform gyrus | 36, -60, -18 | 1013 | <0.001 | 4.28 |
| CVVLT-10 min | Left anterior cingulate cortex | -8, 46, -4 | 1770 | <0.001 | 4.65 |
|  | Right fusiform gyrus | 42, -42, -24 | 1106 | <0.001 | 4.31 |
|  | Superior frontal gyrus | -24, 54, -20 | 372 | 0.032 | 4.47 |
| CVVLT-cued | Right inferior temporal gyrus | 50, -60, -18 | 1223 | <0.001 | 4.61 |
|  | Right middle orbitofrontal gyrus | 28, 48, -14 | 463 | 0.013 | 4.11 |
| **Right entorhinal seed** | |  |  |  |  |
| CVVLT-30 sec | Left inferior orbitofrontal gyrus | -40, 38, -10 | 4159 | <0.001 | 5.01 |
|  | Left inferior temporal gyrus | -62, -36, -18 | 1075 | <0.001 | 4.37 |
|  | Left lingual gyrus | -20, -44, -2 | 424 | 0.015 | 4.80 |
| CVVLT-10 min | Left lingual gyrus | -20, -44, -2 | 2276 | <0.001 | 4.99 |
|  | Left middle orbitofrontal gyrus | -34, 44, -14 | 1979 | <0.001 | 4.73 |
| CVVLT-cued | Left inferior temporal gyrus | -62, -34, -18 | 887 | <0.001 | 4.24 |
|  | Right posterior cingulate cortex | 2, -50, 30 | 655 | 0.001 | 4.32 |
|  | Left inferior orbitofrontal gyrus | -34, 44, -16 | 562 | 0.002 | 4.26 |
|  | Left lingual gyrus | -20, -44, -2 | 292 | 0.041 | 4.63 |
| **Left PCC seed** |  |  |  |  |  |
| CVVLT-30 sec | Left insula | -32, 12, -6 | 442 | 0.008 | 4.49 |
| CVVLT-10 min | Left insula | -32, 12, -6 | 392 | 0.013 | 4.52 |
| CVVLT-cued | Left insula | -26, 12, -14 | 385 | 0.018 | 4.53 |
| **Right PCC seed** |  |  |  |  |  |
| CVVLT-30 sec | Left insula | -32, 12, -6 | 367 | 0.017 | 4.42 |
| CVVLT-10 min | No pick cluster |  |  |  |  |
| CVVLT-cued | Left insula | -26, 12, -14 | 305 | 0.042 | 4.44 |

T maxima, and contiguous voxels of cluster size are shown. The significance clusters are detected with a threshold of FDR-corrected P < 0.05 at cluster-level and uncorrected P < 0.001 at peak-level. CVVLT, Chinese version of the Verbal Learning Test (CVVLT-30 sec: free recall after 30 seconds; CVVLT-10 min: free recall after 10 minutes; CVVLT-cued: recall with cued procedures); DMPFC, dorsal medial prefrontal cortex; FDR, false discovery rates; MNI, Montreal Neurological Institute; PCC, posterior cingulate cortex.
