# Supplementary material for: ABCA7 polymorphisms correlate with memory impairment and default mode network in patients with APOEε4-associated Alzheimer’s disease
Source: Alzheimers Res Ther. 2019 Dec 12;11:103. doi: 10.1186/s13195-019-0563-3 (PMC6909474; doi:10.1186/s13195-019-0563-3)
Supplement: Supplementary file 5 — Additional file 5 : Table S4. Correlations of activity in brain regions in networks anchored by each seed of default mode network with each memory function score. [file 13195_2019_563_MOESM5_ESM.docx]

**Title**

*ABCA7* Polymorphisms Correlate with Memory Impairment and Default Mode Network in Patients with *APOE*ε4 Associated Alzheimer’s Disease

**Journal name**

Alzheimer’s research & therapy

**Author names**

Ya-Ting Chang*^1^ MD, PhD; Shih-Wei Hsu^2^, MD; Shu-Hua Huang^3^ MD; Chi-Wei Huang^1^ MD, PhD; Wen-Neng Chang^1^ MD; Chia-Yi Lien^1^ MD; Jun-Jun Lee^1^ MD; Chen-Chang Lee^2^ PhD; Chiung-Chih Chang*^1^ MD, PhD

^1^Department of Neurology, Institute of translational research in biomedicine, Kaohsiung Chang Gung Memorial Hospital, Chang Gung University College of Medicine, Kaohsiung 83301, Taiwan

^2^Department of Radiology, Kaohsiung Chang Gung Memorial Hospital, Chang Gung University College of Medicine, Kaohsiung, Taiwan

^3^Department of Nuclear Medicine, Kaohsiung Chang Gung Memorial Hospital, Chang Gung University College of Medicine, Kaohsiung, Taiwan

*Ya-Ting Chang and Chiung-Chih Chang are co‐corresponding authors

Submission Type: Article

**Table S4** Correlations of activity in brain regions in networks anchored by each seed of default mode network with each memory function score

| **Seed**  **Memory score** | **Cluster** | **MNI**  **(x, y, z)** | **Cluster size** | **p-FDR**  **of size** | **T** |
| --- | --- | --- | --- | --- | --- |
| **Left DMPFC seed** |  |  |  |  |  |
| CVVLT-30 sec | Right superior medial frontal gyrus | 4, 68, 6 | 3790 | <0.001 | 5.76 |
| CVVLT-10 min | Right inferior frontal gyrus | 54, 24, 22 | 4430 | <0.001 | 5.78 |
| CVVLT-cued | Right inferior frontal gyrus | 54, 22, 22 | 8535 | <0.001 | 6.26 |
| **Right DMPFC seed** |  |  |  |  |  |
| CVVLT-30 sec | No peak cluster |  |  |  |  |
| CVVLT-10 min | No peak cluster |  |  |  |  |
| CVVLT-cued | Left middle frontal gyrus | -42, 28, 40 | 124 | 0.004 | 5.09 |
| **Left entorhinal seed** |  |  |  |  |  |
| CVVLT-30 sec | Right middle orbitofrontal gyrus | 28, 48, -14 | 1922 | <0.001 | 5.11 |
|  | Right fusiform gyrus | 36, -60, -18 | 409 | 0.004 | 4.36 |
| CVVLT-10 min | No peak cluster |  |  |  |  |
| CVVLT-cued | No peak cluster |  |  |  |  |
| **Right entorhinal seed** | |  |  |  |  |
| CVVLT-30 sec | Left inferior orbitofrontal gyrus | -40, 38, -10 | 2349 | <0.001 | 5.08 |
|  | Left inferior temporal gyrus | -62, -36, -18 | 293 | 0.016 | 4.16 |
| CVVLT-10 min | Left middle orbitofrontal gyrus | -34, 44, -14 | 600 | <0.001 | 4.83 |
|  | Left inferior temporal gyrus | -62, -32, -18 | 525 | <0.001 | 4.68 |
| CVVLT-cued | No peak cluster |  |  |  |  |
| **Left PCC seed** |  |  |  |  |  |
| CVVLT-30 sec | No peak cluster |  |  |  |  |
| CVVLT-10 min | No peak cluster |  |  |  |  |
| CVVLT-cued | No peak cluster |  |  |  |  |
| **Right PCC seed** |  |  |  |  |  |
| CVVLT-30 sec | No peak cluster |  |  |  |  |
| CVVLT-10 min | No peak cluster |  |  |  |  |
| CVVLT-cued | No peak cluster |  |  |  |  |

T maxima, and contiguous voxels of cluster size are shown. The significance clusters are detected with a threshold of FDR-corrected P < 0.05 at cluster-level and FDR-corrected P < 0.05 at peak-level. CVVLT, Chinese version of the Verbal Learning Test (CVVLT-30 sec: free recall after 30 seconds; CVVLT-10 min: free recall after 10 minutes; CVVLT-cued: recall with cued procedures); DMPFC, dorsal medial prefrontal cortex; FDR, false discovery rates; MNI, Montreal Neurological Institute; PCC, posterior cingulate cortex.
